# *Trichomonas vaginalis* in bronchoalveolar lavage fluid of a patient with severe pneumonia detected by metagenomic next-generation sequencing: A case report

**DOI:** 10.1097/MD.0000000000035777

**Published:** 2023-11-10

**Authors:** Zhenzhen Li, Jiang Wang, Xuejing Gou, Zhendong Guo, Feng Xu

**Affiliations:** a Department of Pulmonary and Critical Care Medicine, Cangzhou People’s Hospital, Cangzhou, Hebei Province, China.

**Keywords:** bronchoalveolar lavage fluid, metagenomic next-generation sequencing, pulmonary infection, *Trichomonas vaginalis*

## Abstract

**Rationale:**

*Trichomonas vaginalis (T. vaginalis*) is a common anaerobic parasitic protozoan. However, to the best of our knowledge, there are few reports documenting *T. vaginalis* infection outside the genitourinary tract. Severe pneumonia caused by *T. vaginalis* infection has been rarely reported.

**Patient concerns:**

The 80-year-old female patient had a 20-year history of type II diabetes; however, she was not on regular medication. She was hospitalized due to a coma which continued 2 hours caused by trauma after a car accident. After her admission, she was provided with continuous mechanical ventilation; during the ventilation, she was still in a coma, accompanied by repeated fever and presence of much yellow sticky phlegm. The head CT scan indicated temporal lobe hematoma and subarachnoid hemorrhage. The lung CT scan showed bilateral pulmonary inflammatory consolidation and mass lesions.

**Diagnoses:**

She was initially diagnosed with severe pneumonia and acute respiratory distress syndrome. Subsequently, fiberoptic bronchoscopy was conducted, and bronchoalveolar lavage fluid (BALF) was collected and sent for metagenomic next-generation sequencing (mNGS). The result indicated the presence of abundant sequences from the *T. vaginalis* genome. Thus, she was diagnosed with pulmonary *T. vaginalis* infection.

**Intervention:**

Anti-infective ornidazole treatment has significantly improved her symptoms.

**Outcomes:**

After treatment, the patient regained consciousness and was able to communicate, and there was no obvious expectoration, fever, or positive bronchus sign in the lungs. Thereby, she was discharged from the hospital.

**Lessons:**

Special attention should be paid to infections other than common bacterial infections, such as *T. vaginalis.* Moreover, infection of rare pathogenic microorganisms might show symptoms similar to common bacterial infection, leading to misdiagnosis, further highlighting the usefulness of mNGS in detecting pathogens in a timely, sensitive, and accurate manner.

## 1. Introduction

*Trichomonas vaginalis (T. vaginalis*) is a common anaerobic parasitic protozoan that has caused non-viral sexually transmitted infection worldwide.^[1]^ Patients infected with *T. vaginalis* will typically show various symptoms, including vaginal discharge, vaginitis, pruritus, and/or dysuria.^[2,3]^ However, some infected patients do not present typical symptoms. Persistent *T. vaginalis* infections might cause infertility or unfavorable birth outcomes.^[4]^ Moreover, *T. vaginalis* infection is associated with an elevated risk of human immunodeficiency virus infection and cervical cancer development.^[5,6]^ However, to the best of our knowledge, there are few documented cases concerning *Trichomonad* infection in body systems beyond the genito-urinary tract, as *T. vaginalis* has a preference for inhabiting the genitourinary tract and demonstrates specificity for this particular site.^[7]^

Pulmonary *T. vaginalis* infections in adults and children are relatively rare. In fact, pulmonary *Trichomonas tenax* infection has been found mainly in patients with cancer or other lung diseases.^[8]^ For example, Leterrier et al reported the detection of *Trichomonas tenax* in the pleural fluid of a glioblastoma patient with severe pleurisy.^[9]^ As other examples, pulmonary *T. vaginalis* infection has been reported in neonates and adults with respiratory distress.^[10,11]^ Nevertheless, to the best of our knowledge, death caused by pulmonary *T. vaginalis* infection has rarely been reported.^[12]^ Meanwhile, various trichomonas species in clinical cases bring great challenges in the diagnosis of related infectious diseases.

With the rapid development of next-generation sequencing (NGS), DNA or RNA sequencing has become an accurate and effective method for the detection of pathogens.^[13]^ In clinical practice, many rare pathogens in various critical infectious diseases have been identified using NGS. In a recent case presented as severe diffuse alveolar hemorrhage, metagenomic NGS (mNGS) has been proven to be a powerful tool to identify the pathogen (i.e., leptospira) in the bronchoalveolar lavage fluid (BALF).^[14]^ As far as we know, there is currently no report of severe pneumonia caused by pulmonary *T. vaginalis* infection.

The current case report not only provides more reference information for the differential diagnosis of a severe pneumonia case caused by a rare pulmonary *T. vaginalis* infection but also showcases the power of mNGS in clinical applications, especially in identifying atypical pathogens.

## 2. Case report

On October 26, 2021, an 80-year-old woman was admitted to the emergency department of our hospital due to being in a coma for 2 hours, caused by trauma after a car accident. Written informed consent was obtained from the next of kin for the patient. This study was performed in accordance with the Helsinki Declaration and approved by the Ethics Committee of the Cangzhou People Hospital (No. AF/SC-08/02.0). The car accident caused multiple head and limb injuries, unconsciousness, and vomiting in the patients. The head computed tomography (CT) scan indicated temporal lobe hematoma and subarachnoid hemorrhage. The joint CT scan showed multiple fractures on the right tibia and fibula. Subsequently, the subdural hemorrhage and necrotic brain tissues were removed, and an open reduction of fracture was conducted. The patient was provided with continuous mechanical ventilation during the operation, but she remained in a coma after the operation, accompanied by repeated high fever and presence of yellow sticky phlegm. The lung CT scan suggested inflammatory consolidation and mass lesions in bilateral lungs.

The patient had a 20-year history of type II diabetes and had an unstable blood sugar level, but she was not on regular medication. Moreover, she had a 20-year history of coronary atherosclerotic heart disease and took intermittent Suxiao Jiuxin Pills (i.e., a Chinese patent drug mainly composed of Chuan Xiong (Rhizoma Chuanxiong) and Bing Pian (Borneolum), at 40 mg/tablet, produced by Tianjin Zhongxin Pharmaceutical Inc. (Tianjin, China)), which are a quick-acting heart reliever. She had no history of hypertension, infectious disease, or allergy, and no previous personal or family history of *T. vaginalis* infection.

**Figure 1.**
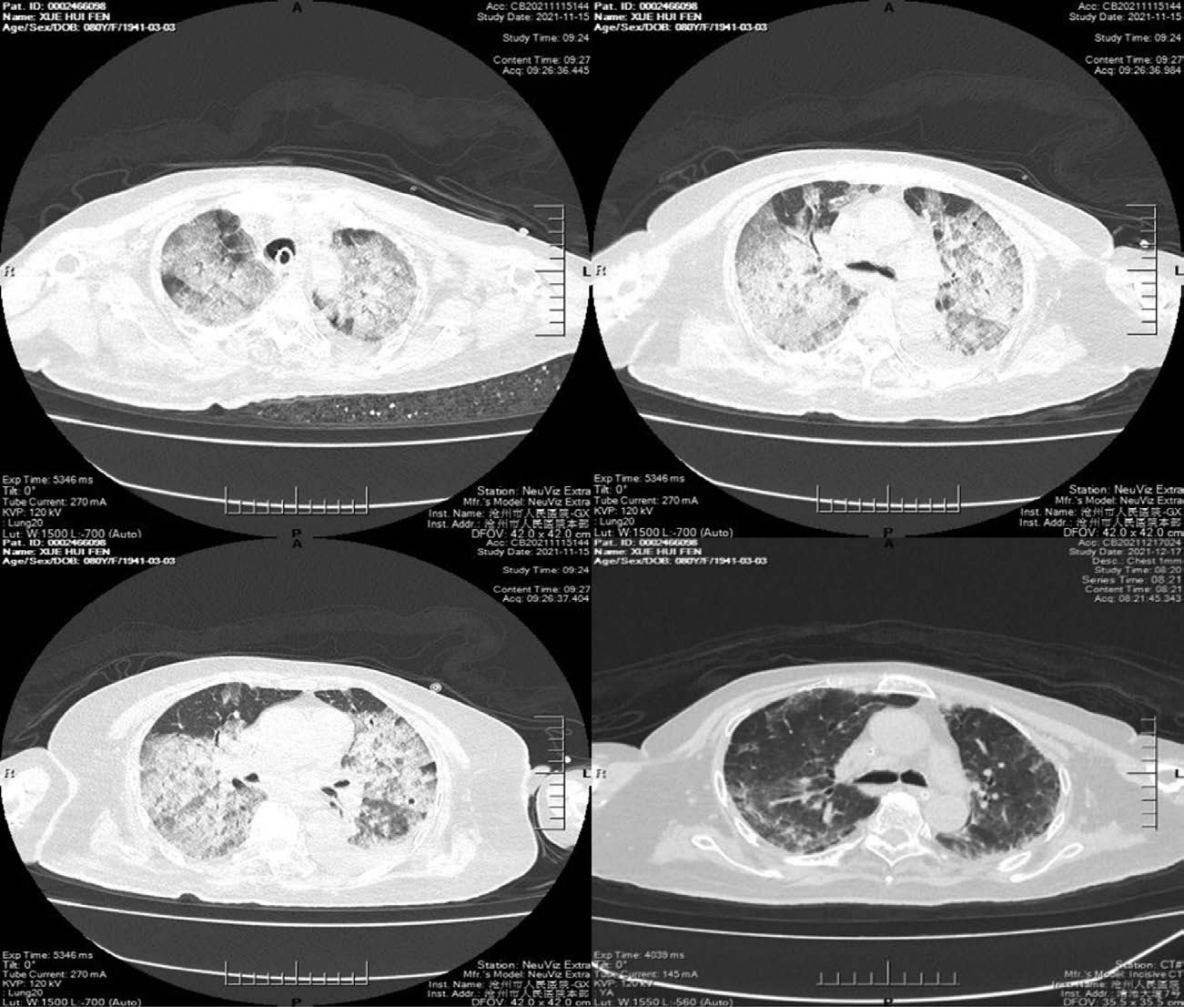
Results of CT scans of the lungs before anti-infective treatment showed the presence of bilateral pneumonia, enlarged pulmonary artery, and left pleural effusion. CT = computed tomography.

After admission, the patient was in a light coma with a Glasgow coma scale of 6, and the oxygen saturation level was 96%, with a mechanical ventilation fraction of inspired oxygen (FiO2) of 60%. Both light and corneal reflexes were sluggish. The right ankle was swollen and deformed, and the bilateral Babinski signs were positive. There were rough sound of breath, wheezing, and coarse rales in bilateral lungs. The white blood cell count was 11.09 × 10^9^/L, neutrophil ratio was 95.4%, hemoglobin level was 100 g/L, and platelet (PLT) count was 164 × 10^9^/L. C-reactive protein level was 219.50 mg/L, procalcitonin level was 1.378 ng/mL, and oxygenation index was 250. Sputum culture indicated *Pseudomonas aeruginosa* infection, and the conventional bacterial culture of BALF was negative. No abnormality was observed by gynecological ultrasound and abdominal and pelvic CT examinations. However, a CT scan of the lungs indicated bilateral pneumonia, enlarged pulmonary artery, and left pleural effusion in the patient (Fig. 1).

As she remained unconscious and had yellow sticky phlegm, we performed a tracheotomy and maintained continuous mechanical ventilation. Meanwhile, intermittent tracheoscopy was performed to facilitate airway management to promote sputum drainage and maintain the patency of the airway. Meropenem was used for anti-infective therapy, but repeated fever (39.0˚C) and the presence of yellow sticky sputum had not been improved significantly. Then, BALF was sent for mNGS; after a strict sequence alignment with the help of a microbiologist, fragments of *T. vaginalis, Candida glabrata, Atopobium vaginae*, and *Gardnerella vaginalis* genomes were identified. The number of detected *T. vaginalis* sequences was 5335. Thus, the patient was diagnosed with severe pneumonia, pulmonary *T. vaginalis* infection, acute respiratory distress syndrome, brain contusion, and traumatic subarachnoid hemorrhage.

**Figure 2.**
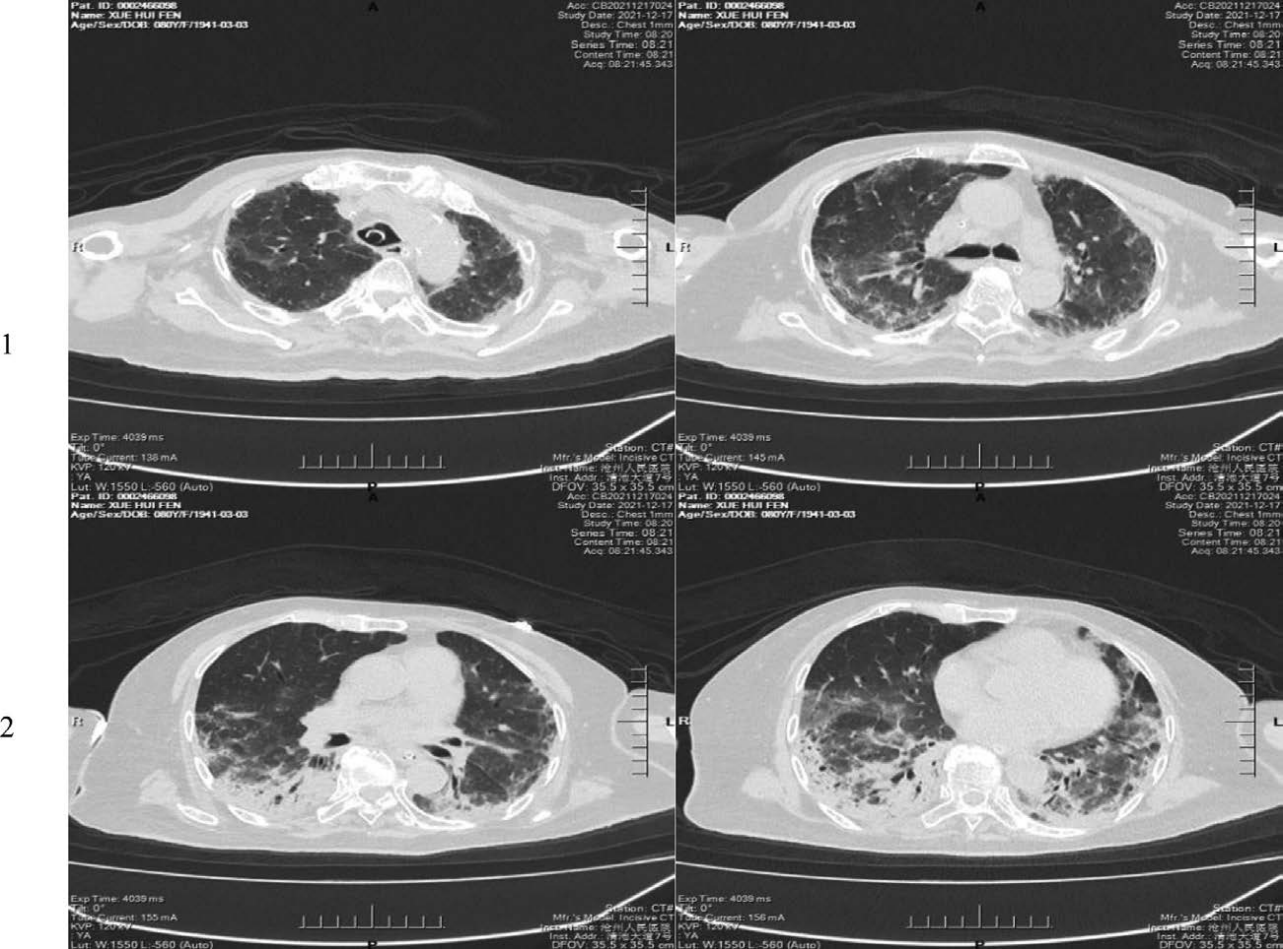
Results of CT scans of the lungs after anti-infective treatment showed that the inflammatory exudation and solid lesions almost completely disappeared. CT = computed tomography.

Previous empirical broad-spectrum antibiotics (Meropenem) treatment showed no effects; however, after the detection of *T. vaginalis* by mNGS, we applied ornidazole and the patient condition was significantly improved. Specifically, after 35 days of treatment with ornidazole injection (once every 12 hours, from November 19, 2021 to December 24, 2021), the patient regained consciousness and was able to communicate, and there was no obvious expectoration, fever, or positive bronchus sign in the lungs. The lung CT scan showed that inflammatory exudation and solid lesions almost completely disappeared (Fig. 2, on December 25, 2021). Thus, the patient was discharged from the hospital.

## 3. Discussion and conclusions

In this case report, the patient was hospitalized due to a 2-hour coma caused by trauma after a car accident. She was diagnosed with severe pneumonia and pulmonary *T. vaginalis* infection. It is extremely rare that *T. vaginalis* was detected in BALF. Further, the detection of *T. vaginalis* in the lungs indicated that more attention should be paid to rare pathogenic microorganisms in critical diseases.

The patient head CT scan indicated temporal lobe hematoma and subarachnoid hemorrhage, and the lung CT scan indicated inflammatory consolidation and mass lesions in bilateral lungs. Accordingly, severe pneumonia and acute respiratory distress syndrome were considered; however, little improvement was observed after treatment with anti-infective therapy. The BALF was sent for mNGS, and there was a high load of *T. vaginalis* sequences, suggesting the patient had a pulmonary *T. vaginalis* infection. Actually, the symptoms and the common physical, laboratory and imaging examination results both suggested bacterial pneumonia. However, since there was no response to empirical broad-spectrum antibiotics (meropenem), and mNGS suggested a *T. vaginalis* infection, ornidazole was started and the patient condition was significantly improved.

As a common parasitic protozoan colonized in the urogenital tract, *T. vaginalis* has been rarely detected outside the genitourinary tract, such as causing severe pneumonia in the lungs.^[15]^ It has been demonstrated that *Trichomonas tenax* (another species of *Trichomonas*) mainly causes bronchopulmonary infections in patients with cancer or other lung diseases.^[8]^ However, pulmonary *T. vaginalis* infection has never been reported in adults. In 2002, Szarka et al documented 2 cases of neonatal pneumonia in which *T. vaginalis* was detected in a newborn tracheal discharge, and subsequently in her mother tracheal discharge as well.^[16]^ These 2 newborn babies suffered from severe congenital breathing difficulties. The microscopic examination and CPLM medium culture finally confirmed the symptom was caused by *T. vaginalis* infection. Whereas in another more recent case (2020) of neonatal pneumonia caused by *T. vaginalis*, the patient also benefited from a 28-day targeted antibiotic therapy after the diagnosis of *T. vaginalis* infection by multiplex real-time PCR technique.^[17]^ Thus, atypical pathogen infections should be considered when the patients do not respond to conventional therapy. Moreover, Salvador-Membreve et al demonstrated that *T. vaginalis* could exert a cytopathic effect on lung alveolar basal carcinoma epithelial cells,^[7]^ supporting the unfavorable impacts of *T. vaginalis* on lungs. *T. vaginalis* infection in the urogenital tract could display distinct phenotypes.^[18]^ However, the current adult patient was an extremely rare case. On the one hand, previous reports showed that pulmonary *T. vaginalis* infection mainly occurred in neonates due to maternal infection. On the other hand, there was no *T. vaginalis* infection in this patient genitourinary tract. She showed severe pneumonia accompanied by repeated fever and presence of much yellow sticky phlegm. This case thus provides valuable reference information on pulmonary *T. vaginalis* infection. Besides, it is quite hard to identify *T. vaginalis* infection without mNGS in this case, highlighting the significance of mNGS as a diagnostic tool for infectious diseases.

Typically, culture-based tests or immunological assays can only detect known pathogens; besides, obvious limitations exist in these detection methods, such as long testing time and lack of sensitivity.^[19]^ In contrast, the rapid development of molecular and mNGS techniques has provided more alternatives for clinical diagnosis and treatments.^[20]^ Recently, the utility, sensitivity, and specificity of mNGS have been widely explored in diagnosing various diseases, including pneumonia.^[21–23]^ Accumulating evidence has indicated the significant advantages of mNGS, including shorter diagnosis time, higher diagnostic sensitivity, and greater capacity in detecting pathogens of different species (e.g., bacteria, viruses, and fungi) compared with traditional methods.^[24]^ For instance, Chen et al showed the effectiveness of mNGS in diagnosing critically ill or immunocompromised patients using BALF samples.^[25]^ In the current case, mNGS revealed sequence fragments of the *T. vaginalis, Candida glabrata, Atopobium vaginae*, and *Gardnerella vaginalis* genomes in the BALF, suggesting infections from these microbes. The mNGS data helped us target causative microorganisms quickly, leading to appropriate intervention, which significantly alleviated the patient symptoms. Our results also highlight the power of mNGS in determining rare pathogens in clinical practice. Nevertheless, the current limitation of mNGS mainly comes from the report interpretation of mNGS data,^[21]^ and thus this method should be combined with clinical symptoms as well as conventional methods. The precise interpretation of mNGS data would be further conducive to the application of mNGS in more clinical cases.

Although we tried to document this rare case in detail, there were still several limitations in our present study. This patient came to our attention after she was diagnosed with *T. vaginalis* infection, thus the clinical information was collected after that. Some clinical information was incomplete or missing, which obviously limited the comprehensive understand of this case.

In conclusion, we herein reported an extremely rare case that the patient was diagnosed with severe pneumonia caused by pulmonary *T. vaginalis* infection. Our case reminded us that special attention should be paid to infections other than common bacterial infections, such as *T. vaginalis.* Moreover, infection of rare pathogenic microorganisms might show symptoms similar to common bacterial infection, leading to misdiagnosis, further highlighting the usefulness of mNGS in detecting pathogens in a timely, sensitive, and accurate manner.

## Author contributions

**Data curation:** Zhenzhen Li, Jiang Wang, Xuejing Gou, Zhendong Guo, Feng Xu.

**Writing – original draft:** Zhenzhen Li.

**Writing – review & editing:** Feng Xu.
